# Case report: Gastroenterological management in a case of cardio-facio-cutaneous syndrome

**DOI:** 10.3389/fped.2023.1160147

**Published:** 2023-04-17

**Authors:** B. Ciacchini, G. Di Nardo, M. Marin, E. Borali, M. Caraccia, R. Mogni, F. Cairello, I. Rabbone, G. B Ferrero, A. Pini Prato, E. Felici

**Affiliations:** ^1^ Division of Pediatrics, Department of Health Science, University of Piemonte Orientale, Novara, Italy; ^2^ NESMOS Department, Faculty of Medicine and Psychology, Sant'Andrea University Hospital, Sapienza University of Rome, Rome, Italy; ^3^ Pediatric and Pediatric Emergency Unit, “U. Bosio” Center for Digestive Diseases, The Children Hospital, AO SS Antonio e Biagio e C. Arrigo, Alessandria, Italy; ^4^ Pediatric Surgery, “U. Bosio” Center for Digestive Diseases, The Children Hospital, AO SS Antonio e Biagio e C. Arrigo, Alessandria, Italy; ^5^ Department of Clinical and Biological Sciences, University of Torino, Turin, Italy

**Keywords:** pediatric, gastroenterology, cardio-facio-cutaneous syndrome (CFCS), nutrition, surgery

## Abstract

**Background:**

cardio-facio-cutaneous syndrome is a rare genetic disorder affecting less than 900 people in the world. It is mainly characterized by craniofacial, dermatologic and cardiac defects, but also gastroenterological symptoms may be present, ranging from feeding difficulties to gastroesophageal reflux and constipation.

In this report we describe a case of this syndrome characterized by severe feeding and growth difficulties, with a particular focus on the management of gastroenterological complications.

**Case presentation:**

the patient was a caucasian male affected by Cardio-Facio-Cutaneous syndrome who presented feeding difficulties already a few hours after birth. These symptoms worsened in the following months and lead to a complete growth arrest and malnutrition. He was first treated with a nasogastric tube placement. Subsequently, a laparoscopic Nissen fundoplication and a laparoscopic Stamm gastrostomy were performed. The child was fed with nocturnal enteral nutrition and diurnal oral and enteral nutrition. Eventually the patient resumed feeding validly and regained adequate growth.

**Conclusion:**

this paper aims to bring to light a complex rare syndrome that infrequently comes to the attention of the pediatricians and whose diagnosis is not always straightforward. We also highlight the possible complications under a gastroenterologic point of view. Our contribution can be helpful to the pediatrician in the first diagnostic suspect of this syndrome. In particular, it is worth highlighting that -in an infant with Noonan-like features- symptoms like suction or swallowing problems, vomiting and feeding difficulties should orient towards the diagnosis of a Cardio-facio-cutaneous syndrome. It is also important to stress that its related gastroenterological issues may lead to severe growth failure and therefore the role of the gastroenterologist is key to manage supplemental feeding and to establish whether a nasogastric or gastrostomic tube placement is necessary.

## Background

Cardio-facio-cutaneous syndrome (CFCS) (OMIM 115150) is a rare genetic syndrome belonging to the group of Rasopathies. It is caused by a dysregulation of the Ras/MAPK pathway arising from heterozygous germline activating mutations in BRAF (∼75%), MEK1 and MEK2 (∼25%) or KRAS (<2%) protein kinases (1, 2). Usually *de novo* mutations are identified in these patients (1, 2). It is currently possible to estimate less than 900 patients with CFCS worldwide (3). The clinical diagnosis is not easy, especially during the first months of life, due to the overlapping features with other Rasopathies, such as Noonan and Costello syndrome (1, 4, 5). The diagnostic approach should rely on next generation sequencing analysis of the Rasopathies gene panel, in order to confirm the clinical suspicion and correctly define prognosis and management. The syndrome is mainly characterized by craniofacial, dermatologic and cardiac defects (1), but it may also present gastroenterological symptoms, ranging from feeding difficulties to gastroesophageal reflux and constipation, with consequent growth difficulties or arrest (1). In this report we describe a case of CFCS due to a *de novo* mutation of the BRAF gene (c.1914T > G) with a particular focus on the management of gastroenterological complications.

## Case presentation

The patient was a caucasian male, first-born child of healthy, non-consanguineous parents. He was born at 37 weeks of gestation to a *primigravida* mother with normal pregnancy and delivery. Prenatal karyotype was 46, XY. His birth weight was 3230 g (78th percentile), length 50 cm (79th percentile) and head circumference 35.5 cm (94th percentile). He presented craniofacial dismorphism, including tall forehead with bitemporal narrowing, low set ears and small chin, therefore whole exome sequencing was requested. He also had a 3.5 cm congenital melanocytic nevus in the right epigastric region. A few hours after birth he presented feeding difficulties with uncoordinated suction and tendency to vomit. For these reasons an abdomen ultrasound and an x-ray with contrast medium of the gastrointestinal tract were performed and revealed a normal morphology and motility of the digestive tract. Blood tests demonstrated normal liver, kidney and thyroid function. Plasma and urinary amino acid analysis and the metabolic work-up excluded a metabolic disease. He was discharged with the prescription of an anti-reflux milk and glucolipid supplementation.

Subsequently, child's feeding difficulties worsened and he started to present food aversion, leading to a profound failure to thrive. Gastroesophageal reflux therapy with omeprazole was attempted with no benefit. The patient was admitted to our clinic for the first time at age of 7 months due to his growth arrest (his weight was <3_rd_ percentile). He presented relative macrocephaly, sparse and curly hair, hypertelorism, broad nasal base and small chin.

Abdomen ultrasonography as well as esophago-gastro-duodenoscopy were normal. An evaluation by a deglutition specialist was performed and found to be normal, except for food aversion. Since symptoms persisted despite the ongoing pharmacological therapy, 24h pH-impedance monitoring on-therapy was performed, showing a significantly high number of alkaline reflux episodes, while it turned out negative for acid gastro-esophageal reflux. In consideration to malnutrition, nasogastric tube was placed, and he started a continuous nocturnal enteral nutrition with a hyper caloric milk, plus three semi-liquid meals during the day. Omeprazole therapy was continued for another month, then discontinued. Enteral nutritional therapy resulted in a good weight recovery, while the frequent postprandial and nocturnal vomiting persisted.

Our gastroenterological team, after an in-depth interview with patient's parents and multidisciplinary discussion of the whole clinical picture, decided to perform a laparoscopic Nissen fundoplication along with a laparoscopic Stamm gastrostomy, which was performed when the child was 11 months old. The child was being fed with a with a hysocaloric formula (1 kcal/ml) supplied during a cycle of nocturnal enteral nutrition and diurnal oral nutrition, with the addiction of boluses of enteral nutrition when the oral intake was insufficient to reach the prescribed caloric amount. Before surgery the caloric intake provided to the child was 500 kcal/day, while after it was 900 kcal/day, adequate to satisfy his nutritional needs.

**Figure 1:**
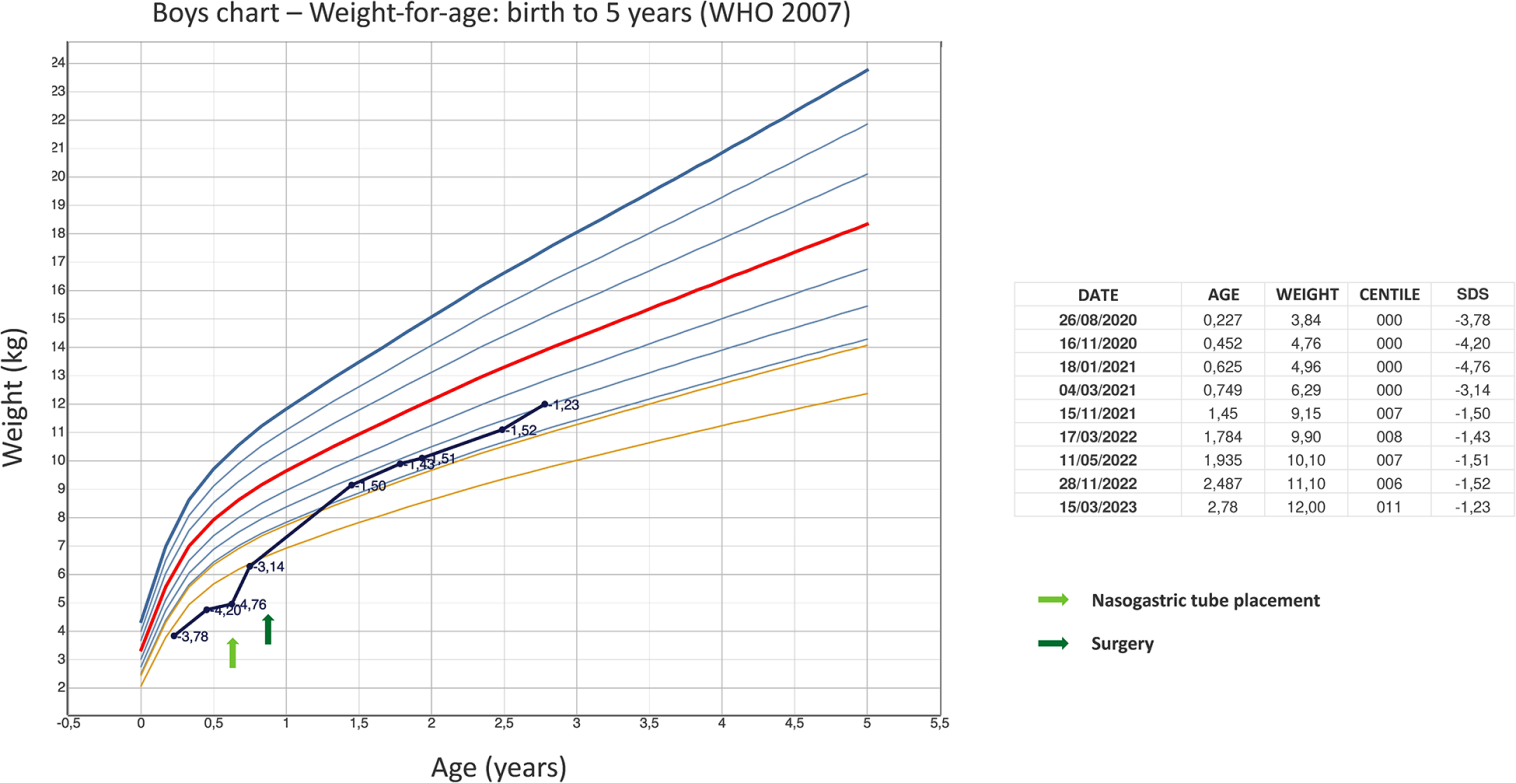
The graph shows the child's weight improvement reported on the WHO boys charts—weight-for-age: birth to 5 years. Arrows indicate times when nasogastric tube was placed and surgery was performed.

The patient is currently 2.5 years old, he is still fed mostly *via* gastrostomy tube, and has reached a weight corresponding to about the 10th percentile (Figure 1), even though a severe food aversion is still present. Therefore, rehabilitation with a deglutition specialist is ongoing.

Around the same time the surgery was performed, genetic tests revealed the pathogenetic variant c.1914T > G (p.Asp638Glu) in heterozygosity in the BRAF gene, a *de novo* mutation in the patient. This variant has already been described in patients with CFCS (6).

Given the diagnosis, genetic counseling was performed, and a multidisciplinary follow-up was set up, according to the specific guidelines for the syndrome. He underwent brain MRI, which revealed a cerebellar vermis of dimensions at the lower limits, although not hypoplastic. This finding, which to our knowledge is not typical of CFCS, is still being followed up and might not be confirmed in subsequent instrumental examinations. The child shows a delay in the developmental stages, and undergoes neuropsychiatric and physiotherapic follow-ups regularly. From a cardiological point of view, he has never presented any issue, and ECG and echocardiography are currently normal. He is also in a dermatological follow-up for the congenital melanocytic nevus, already described in the literature as associated with the mutation of the BRAF gene (6), which has been considered by dermatologists to have a low risk of malignant evolution.

## Discussion and conclusion

At the age of 9 months, shortly after the diagnosis of CFCS, our patient was in fair general condition. He presented a delay in the developmental stages and a poor weight growth, strongly worsened by feeding difficulties.

The gastroenterological issue, representing the main and most disabling problem of our patient, is one of the aspects that more often affect patients with CFCS. There are many pieces of evidence in literature about the gastroenterological problems of CFCS patients: during infancy, severe feeding difficulties are common, including gastroesophageal reflux, suction or swallowing difficulties, aspiration, vomiting, and oral aversion (1, 2). Early signs of swallowing difficulties may be polyhydramnios and, after birth, the difficulty in maintaining an adequate caloric intake with oral feeding (7, 8).

In fact, in an infant with Noonan-like facial features, when the diagnosis is not yet known, severe feeding difficulties, gastroesophageal reflux and failure to growth should suggest the possible diagnosis of a CFCS. In many cases gastroenterological issues contribute to poor growth or growth failure (1, 2), often characterized by weight below the 3rd centile, associated with short stature and relative macrocephaly (1).

Due to failure to thrive, about half of the children with CFCS require nasogastric or gastrostomy tube feeding (1, 2, 7) and there are frequent cases of prolonged supplemental feedings through this type of support, which can persist up to late childhood and adolescence (9). Respiratory complications may also occur, such as choking, aspiration pneumonia, and chronic raspy breathing, related to the swallowing problems (1, 7).

An increase in calorie intake may be helpful in promoting growth of these children. Of note, feeding therapy is suggested at the first sign of oral aversion. As many CFCS patients experience severe gastroesophageal reflux, some suggested the use of maximal medical treatment or even surgery as reported in the present case (1).

Feeding difficulties usually improve in late childhood (2), while oral aversion and sensory integration difficulties with solid foods may persist into adulthood (1). Furthermore, constipation, related to intestinal dysmotility, is frequently described and can represent a lifelong problem (1, 2).

Other gastrointestinal problems, such as intestinal malrotation, functional megacolon, anal stenosis, antral foveolar hyperplasia, are reported with lower frequency (1, 2, 7). In some cases, splenomegaly or hepatomegaly and fatty liver have also been reported (1, 2).

Based on literature and on our case, children with known or suspected CFCS, should undergo early pediatric management in order to strictly monitor thrive and to promptly identify possible feeding difficulties or gastroesophageal reflux. The role of the gastroenterologist is pivotal to decide whether supplemental feeding is necessary, to manage severe feeding problems and to establish if a nasogastric or gastrostomic tube placement is necessary.

This paper aims to cast light on a complex, rare syndrome that infrequently comes to the attention of the pediatrician and that is not easy to diagnose. We also highlight the possible complications from a gastroenterologic point of view. Our contribution can aid the pediatrician in the first diagnostic suspicion of this syndrome. In particular, it is noteworthy that in an infant with Noonan-like features, suction or swallowing problems, any vomiting and feeding difficulties should suggest the diagnosis of a Cardio-facio-cutaneous syndrome. It can also be useful to consider that gastroenterological issues may lead to severe growth failure, therefore the role of the gastroenterologist is key to manage supplemental feeding and to establish if a nasogastric or gastrostomic tube placement is necessary.

As a matter of fact, consensus guidelines for surveillance in CFCS establish that, if anomalies are identified in any organ system, periodic follow-up is indicated to these patients for life ( 1 ).

In conclusion, CFC is a complex rare syndrome which require a synergic multidisciplinary management with specialists care, not only for the patient but also for the whole family. From a gastroenterological point of view, a thorough evaluation upon diagnosis and a long-term follow-up is mandatory.

## Data Availability

The original contributions presented in the study are included in the article/Supplementary Material, further inquiries can be directed to the corresponding authors.
